# Nontoxic Targeting of Energy Metabolism in Preclinical VM-M3 Experimental Glioblastoma

**DOI:** 10.3389/fnut.2018.00091

**Published:** 2018-10-05

**Authors:** Zachary M. Augur, Catherine M. Doyle, Mingyi Li, Purna Mukherjee, Thomas N. Seyfried

**Affiliations:** Thomas N. Seyfried Laboratory, Biology Department, Boston College, Chestnut Hill, MA, United States

**Keywords:** oxaloacetate, temozolomide, ketogenic diet, hyperbaric oxygen therapy, cancer

## Abstract

**Introduction:** Temozolomide (TMZ) is part of the standard of care for treating glioblastoma multiforme (GBM), an aggressive primary brain tumor. New approaches are needed to enhance therapeutic efficacy and reduce toxicity. GBM tumor cells are dependent on glucose and glutamine while relying heavily on aerobic fermentation for energy metabolism. Restricted availability of glucose and glutamine may therefore reduce disease progression. Calorically restricted ketogenic diets (KD-R), which reduce glucose and elevate ketone bodies, offer a promising alternative in targeting energy metabolism because cancer cells cannot effectively burn ketones due to defects in the number, structure, and function of mitochondria. Similarly, oxaloacetate, which participates in the deamination of glutamate, has the potential to reduce the negative effects of excess glutamate found in many brain tumors, while hyperbaric oxygen therapy can reverse the hypoxic phenotype of tumors and reduce growth. We hypothesize that the combinatorial therapy of KD-R, hyperbaric oxygen, and oxaloacetate, could reduce or eliminate the need for TMZ in GBM patients.

**Methods:** Our proposed approach for inhibiting tumor metabolism involved various combinations of the KD-R, oxaloacetate (2 mg/g), hyperbaric oxygen, and TMZ (20 mg/kg). This combinatorial therapy was tested on adult VM/Dk mice bearing the VM-M3/Fluc preclinical GBM model grown orthotopically. After 14 days, tumor growth was quantified via bioluminescence. A survival study was performed and the data were analyzed and portrayed in a Kaplan Meier plot. Preliminary dosage studies were used and strict diet and drug administration was maintained throughout the study.

**Results:** The therapeutic effect of all treatments was powerful when administered under KD-R. The most promising survival advantage was seen in the two groups receiving oxaloacetate without TMZ. The survival of mice receiving TMZ was diminished due to its apparent toxicity. Among all groups, those receiving TMZ had the most significant reduction in tumor growth. The most powerful therapeutic effect was evident with combinations of these therapies.

**Conclusion:** This study provides evidence for a potentially novel therapeutic regimen of hyperbaric oxygen, oxaloacetate, and the KD-R for managing growth and progression of VM-M3/Fluc GBM.

## Introduction

Glioblastoma multiforme (GBM) or grade IV astrocytoma is a rapidly proliferating and invading glioma composed of a heterogeneous population of cell types, and it remains largely unmanageable (1, 2). Temozolomide (TMZ), a toxic alkylating agent, is one of the most commonly used chemotherapeutic drugs to combat GBM (3). Despite its wide use in the clinic, TMZ's most common side effects include hematological toxicity and gastrointestinal troubles, with nausea, vomiting, and anorexia being most common in patients (4). Upon diagnosis, and with the subsequent standard of care, GBM patients have an expected survival of about 12 to 15 months with essentially no long-term survivors (5). A therapy that reduces or eliminates toxicity, while increasing efficacy is therefore necessary for the well-being and survival of the patient.

As previously described, VM-M3 is a syngeneic murine tumor model that closely mimics the phenotype of GBM in humans and was used in this study to test various GBM treatment combinations. Although a single GBM model may not be representative of all heterogeneous cell types in human GBM, we chose the VM-M3 model because, to our knowledge, it is the only natural syngeneic model that manifests all the invasive growth characteristics seen in human GBM and has been shown to exhibit the full spectrum of “Secondary Structures of Scherer” (6, 7). The VM-M3 murine model also exhibits abnormalities in cardiolipin that underlie defects in oxidative phosphorylation (8, 9). It is important to note that oxygen consumption is not always coupled to ATP production and therefore says little of the efficiency of mitochondrial oxidative phosphorylation (10–13). Cardiolipin is the signature phospholipid found in the inner mitochondrial membrane that regulates mitochondrial enzyme activity related to oxidative phosphorylation (14). Cardiolipin abnormality results in electron transport chain activity reduction and an increase in the production of reactive oxygen species via the prevention of coenzyme Q couple oxidation (15). Furthermore, the presence of aerobic fermentation in the VM-M3 tumor model has been established previously (16).

The Press-Pulse approach to cancer management provides a useful model for utilizing metabolic therapies in combatting cancer (17). Press therapies, like restricted feeding of ketogenic diets (KD-R), reduce available glucose while increasing ketone bodies. Ketone bodies can replace glucose as a fuel for cells with normal mitochondrial function. Cancer cells, however, cannot metabolize ketone bodies for energy due to multiple defects in the number, structure, and function of their mitochondria. The KD-R reduces levels of glucose and substrates for both the glycolytic and the pentose phosphate pathways. As a result, cellular energy is reduced and the synthesis of downstream precursors, like glutathione, are hindered. Cancer cells rely heavily on the aerobic fermentation of glucose, which explains the importance of the KD-R in combating cancer (18–22). If administered properly, the KD-R can have extensive health benefits. However, it is important to note that diabetic ketoacidosis is a dangerous complication in patients with type I diabetes. In ketoacidosis, the acids 3-hydroxybutyric acid and acetoacetic acid are produced, which overwhelm the body's acid-base buffering system leading to a variety of health complications. If the KD-R is consumed and an appropriate GKI is monitored and maintained, the regulated and controlled production of ketone bodies causes a beneficial physiological state called dietary ketosis. In this state, the blood pH remains buffered within normal limits (23).

This KD-R press therapy is used to prime tumor cells for more acute pulse therapies that further target glucose and glutamine (17). One potential pulse therapy includes oxaloacetate (OAA). This compound is an important intermediate in the citric acid cycle, but has also been shown to participate in the deamination of glutamate and to serve as a mitochondrial biogenesis enhancer (24, 25). At appropriate levels, Glutamate (Glu) serves as the major excitatory neurotransmitter in the nervous system and is released from synaptic vesicles upon introduction of calcium. In GBM patients, an excess amount of Glu is released, which enhances the already malignant phenotype (3, 26–28). The continuous buildup of Glu in the extracellular environment provides resistance to apoptosis and enhances proliferation and invasion of the GBM cells (29). Further studies observed that glutamate secretion by neoplastic glia promotes tumor expansion by enhancing the inflammatory response within tumor surroundings (30). As shown previously, OAA, may be used via glutamate oxaloacetate transaminase to enhance the naturally occurring brain to blood efflux of Glu. This has been shown to enhance neuro-protection, reduce the negative prognosis of the GBM patient, and serve as an adjuvant for TMZ (24, 31). In both human consumers and preclinical models, OAA has exhibited little toxicity or negative side effects, if administered at an appropriate dose. In fact, along with its potential anti-tumor effects, there is evidence to suggest that OAA can enhance lifespan and overall health (32).

We predicted that the use of Hyperbaric Oxygen Therapy (HBO_2_T), as another pulse therapy, would further enhance the cocktail treatment via increased plasma oxygen saturation. HBO_2_T involves administration of 100% oxygen under pressure (2.5 ATM abosolute), and has been shown to inhibit tumor growth and reduce blood vessel density (33). The increased oxygen saturation facilitates oxygen delivery to the tissues and reduces the hypoxic microenvironment (34). It also enhances tumor-cell production of reactive oxygen species (ROS), which contributes to redox stress and subsequent cancer cell death. While under KD-R, normal cells transition to ketone body metabolism and achieve therapeutic ketosis, which provides protection from ROS and oxidative stress (35, 36). The appropriate use and administration of HBO_2_T provides little risk to the patient. Yet, if left unmonitored, HBO_2_T has the potential to cause mild barotrauma, pulmonary oxygen toxicity, and ocular side effects (37). However, it is important to note that 2.5 ATM absolute is within the therapeutic range of pressures for mice and has been observed to show little negative side-effects. Therapeutic efficacy in both mice and humans is observed when oxygen partial pressures within the body exceed the partial pressure at normal atmospheric pressure. This enhanced oxygen partial pressure in mice under 2.5 ATM absolute HBO_2_T has been shown experimentally (38, 39). With the nontoxic treatments of KD-R, HBO_2_T, and OAA working simultaneously in GBM patients (+/- TMZ), it was predicted that the overall prognosis and survival would significantly increase.

## Materials and methods

### Mice and experimental glioblastoma multiforme model

Mice of the VM/Dk (VM) strain were obtained as a gift from H. Fraser (University of Edinburgh, Scotland) and were housed and bred in the Boston College Animal Facility as previously described (40). Adult male and female mice (10–12 weeks of age) were used for the studies and the procedures for animal use were in strict accordance with the NIH Guide for the Care and Use of Laboratory Animals and were approved by the Institutional Animal Care Committee at Boston College under assurance number A3905-01. The protocol was approved by the Institutional Animal Care Committee at Boston College. The VM-M3 tumor used in this study arose spontaneously in the cerebrum of adult VM mice and was characterized as previously described (6).

### Transduction of cell lines and bioluminescent imaging

The VM-M3 cell line was transduced with a lentivirus vector containing the firefly luciferase gene under the control of the cytomegalovirus promoter (VM-M3/Fluc) as we previously described (41). The Xenogen IVIS system (Xenogen, Hopkington, MA) is used to record the bioluminescent signal from the VM-M3/Fluc tumor cells (VM-M3). For *in-vivo* imaging, mice were anesthetized with 5% isoflurane vapor and received an intraperitoneal injection of d-Luciferin (50 mg/kg, Syd Labs) in PBS. Imaging times ranged from 1 to 5 min. For *ex-vivo* imaging, brains were removed and the right and left cortex were separated at the medial longitudinal fissure. Each section was imaged in 0.3 mg d-Luciferin in PBS and bioluminescent values were combined for whole brain bioluminescent data.

### Intracranial tumor implantation

Tumor implantation is performed as previously described (40, 42, 43). Briefly, mice are anesthetized with 5% isoflurane vapor and the hair on the top of the head is removed. The skin is then disinfected with ethanol followed by betadine. A small sagittal incision is made in the scalp of the mouse over the midline. 1.0 mm^3^ tumor tissue fragments of VM-M3 tumor are implanted surgically into the right cerebral cortex according to our standard procedures. The flaps of skin are then closed with 7 mm reflex clips. It is important to mention that tissue fragments were chosen because they contain an already established tumor microenvironment that increases tumor “take” and rapid tumor growth.

### Dietary regimens and body weight

All mice received the standard diet unrestricted (SD-UR) prior to initiation of the study. Upon implantation of the tumor, mice remained on the SD-UR until a 15-h fast was initiated 72 h after implantation. Following the fast, mice were introduced to the Ketogenic diet restricted, or remained on the SD-UR, depending on the study group. The KD-R was made fresh weekly by combining dry KetoGEN powder with dH2O in a 2:1 ratio. The KD-R has a caloric density of 7.12 Kcal/g. The percent nutritional breakdown of KetoGEN is as follows: 2.1% of Cal from carbohydrates, 8.7% of Cal from protein, and 89.2% of Cal from fat. Mice were individually housed beginning at the 15 h fast and food intake for the KD-R mice was measured to be 1–3 g per day to maintain a 12–15% body weight reduction. Mice were weighed daily to ensure weight maintenance (44).

### Measurement of plasma glucose, β-hydroxybutyrate, and glucose ketone index

Blood was collected from mice on the last day of the experiment to reduce endpoint value variability. This procedure was in accordance with IACUC's recommendation for blood volume collection over a 2-week period. All mice were fasted for 2 h before blood collection to stabilize glucose levels. The mice were anesthetized with isoflurane, and the blood was collected from the submandibular facial vein. The blood was centrifuged at 6,000 rpm for 10 min, and the plasma was collected and stored at −80°C until assayed. Plasma glucose concentration was measured spectrophotometrically using the Trinder Assay (Sigma-Aldrich, St. Louis, MO, USA). Plasma β-hydroxybutyrate concentration was measured using the Sigma-Aldrich Ketone Body Assay Kit (MAK134) (Sigma-Aldrich, St. Louis, MO) (45). The Glucose Ketone Index (GKI) was calculated by dividing values of glucose (mM) by values of β-hydroxybutyrate (mM) (46).

### Drug preparation and administration of therapies

The KD-R was initiated on day 4 after tumor cell implantation into the brain. As OAA (Terra Biological LLC, San Diego, CA) is unstable in solution, we administered OAA to the mice daily in the diet (2.0 mg/g body wt) beginning on day 5 (25). TMZ (20 mg/kg) was administered via an intraperitoneal injection, every other day beginning on day 5, in a 10% DMSO, 90% PBS vehicle, as described previously (47). Mice were injected with TMZ (Sigma-Aldrich, St. Louis, MO) 2 h following the KD-R feeding, and were then placed into the hyperbaric oxygen chamber. Control mice received the vehicle only.

### Hyperbaric oxygen therapy

Mice were placed into a hyperbaric chamber and received 100% O_2_ for 90 min at 2.5 ATM absolute 3x/week as previously described (35).

### Statistical analysis

The one-way analysis of variance (ANOVA) followed by Tukey's *post hoc* test was used to analyze tumor growth bioluminescence and average body weight loss among study groups. Average blood measurements were analyzed by one-way ANOVA with Kruskal Wallis Test and Dunn's Multiple Comparison Test *post hoc*. In each figure, n designates the number of individual mice analyzed. Error bars in the figures are expressed as mean ± SEM. Survival was computed and plotted according to the nonparametric Kaplan-Meier analysis, and comparison of control and treated groups was made using the log-rank test (48). A student's *t*-test that compared either the average bioluminescence or average survival between genders in each group was performed. There was no statistical difference between gender in any study group (*p* > 0.05).

## Results

**Figure 1:**
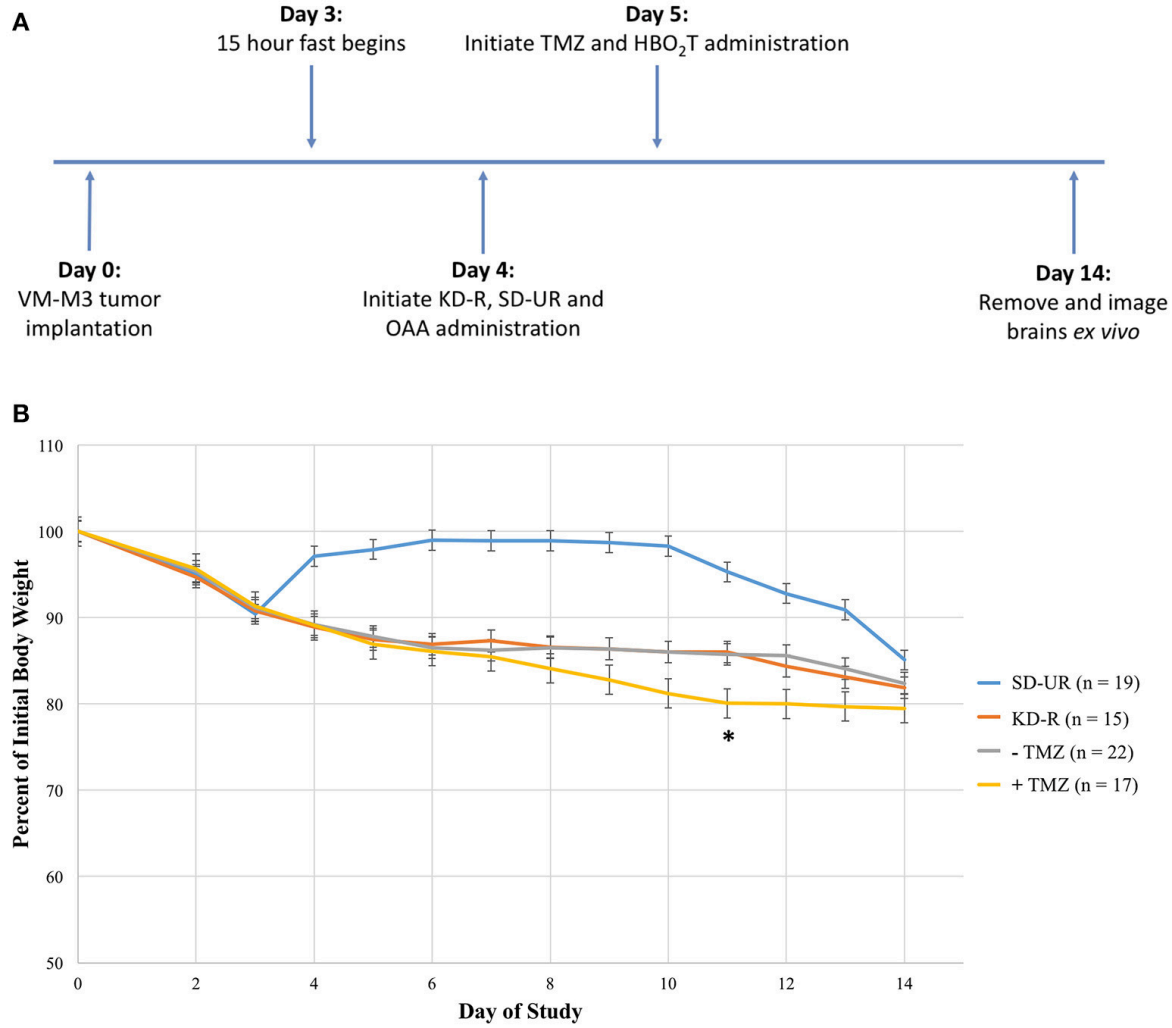
Experimental design and effect of calorie restriction and treatments on mouse body weight. **(A)** Mice were implanted on day 0 with tissue fragments of the VM-M3 glioblastoma and given 3 days to recover before the initiation of KD-R. OAA was administered in the food and prepared fresh daily, as described in the materials and methods. TMZ and HBO_2_T were administered the following day as described in the materials and methods. The experiment was terminated at day 14 when an average number of mice under the SD-UR showed signs of morbidity. In each experiment, SD-UR mice had an average life span of approximately 12–15 days following tumor implantation. Brains were removed and imaged *ex vivo* to measure bioluminescence. **(B)** The body weight of mice receiving SD-UR recovered after the 15 h fast on day 3, but began to decline by day 10 due to tumor burden. The KD-R group and all groups that did not receive TMZ treatment (– TMZ) maintained a 12–15% body weight reduction throughout the study. The mice receiving TMZ treatment (+ TMZ) experienced a 20% body weight reduction by day 11. On day 11, there was a significant difference in body weight (^*^) between the –TMZ group and the +TMZ group (*P* < 0.05). On days 10 and 12, there was a trend toward significance between these two groups.

The objective of this study was to determine if a therapeutic strategy involving a combination of the KD-R, oxaloacetate, hyperbaric oxygen, and temozolomide would be better for managing the VM-M3 mouse glioblastoma than would using the individual therapies alone. We evaluated the dosage, scheduling, and timing needed to achieve the most favorable therapeutic response. It was determined in preliminary studies that 2 mg/g of OAA and 20 mg/kg of TMZ were significantly more efficacious than low dose for both treatments (1 mg/g of OAA and 10 mg/kg TMZ; Supplementary Figure 1). The scheduling and timing was based on these preliminary studies and on the observed VM/Dk mouse response to each treatment. Following our experimental design, we measured tumor growth via bioluminescent imaging (Figure 1A). Due to the rapid growth of the VM-M3 tumor, and the short window of drug treatment (~9 days), the benefits of taking multiple *in vivo* bioluminescent images throughout the study did not justify the inherent risk of potentially losing or altering the endpoint values. Therefore, only one bioluminescent reading was taken at the end of the tumor growth study. The termination day occurred when the average body weight loss of SD-UR mice exceeded 1–1.5 grams per day (5% body weight loss per day). Therefore, the termination day from study to study ranges from day 13 to day 15 with an average being day 14. Additionally, the body weights of all KD-R mice were monitored daily to ensure that a 12–15% body weight reduction was maintained during the course of the study (Figure 1B). A statistically significant average body weight reduction was observed at day 11 in the mice receiving TMZ in comparison to all other groups. This weight reduction was due in part to reduced daily consumption of the KD-R, indicating potential toxicity.

**Figure 2:**
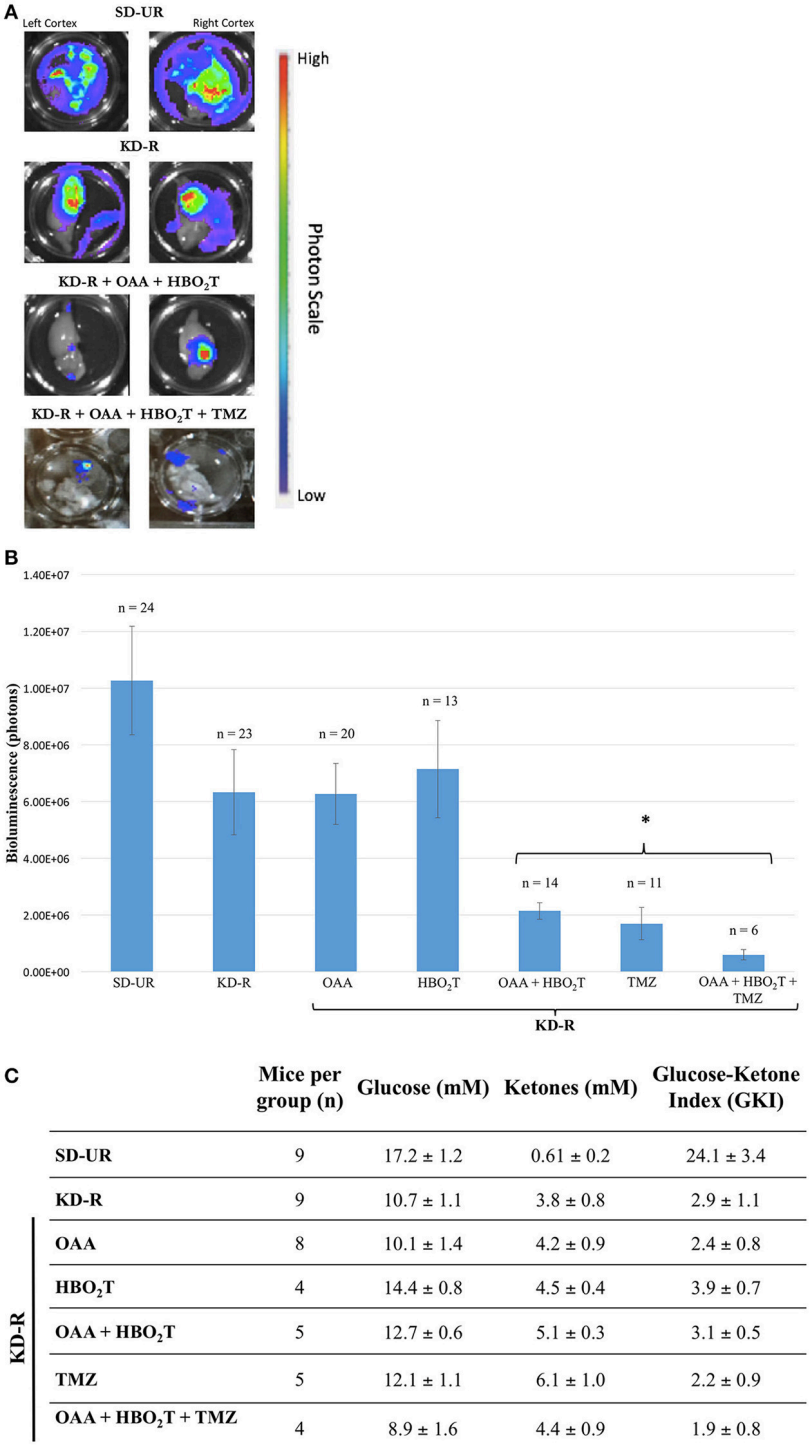
Influence of KD-R, OAA, HBO_2_T, and TMZ on VM-M3/Fluc brain tumor growth and mouse GKI. **(A)** The dissected right and left cortex of the mouse brain were imaged for bioluminescent quantification. Bioluminescence in the contralateral hemisphere is evidence for distal tumor spread. Bioluminescent photon values for the right and left cerebral cortex were combined to determine total brain bioluminescence. **(B)** Whole brain *ex-vivo* bioluminescent data collected from three studies were combined. A significant therapeutic benefit (^*^) for the study groups receiving TMZ was observed when compared to all previous groups receiving individual treatment (*P* < 0.05). The group receiving OAA and HBO_2_T under a KD-R also had a significant reduction in bioluminescence when compared to the four previous groups receiving individual therapies (*P* < 0.05). There was no statistical difference between the two groups receiving TMZ or KD-R + OAA + HBO_2_T. **(C)** Plasma glucose and β-hydroxybutyrate (ketones) values were measured spectrophotometrically from plasma collected from mice on the last day of the experiment, as described in the Materials and Methods. GKI values were calculated for each animal by dividing glucose concentration by ketone concentration, as described. Values represent the mean ± SEM. All groups are statistically different from SD-UR (*p* < 0.05). No group under the KD-R are statistically different from each other.

The first set of experiments involved the bioluminescent quantification of mice receiving combinations of KD-R, HBO_2_T, and OAA, as an adjuvant with or without TMZ. Bioluminescent quantification was obtained via the imaging of the right and left cortex, as described in the materials and methods. Bioluminescence in the left, contralateral hemisphere, is evidence of distal tumor spread in the brain (Figure 2A). The two groups receiving TMZ were included to depict the standard of care administered in conjunction with our proposed nontoxic treatment. The tumor bioluminescence in the groups receiving TMZ was significantly lower than that observed in all other groups receiving individual therapies. The group receiving OAA and HBO_2_T under a KD-R also had a significant reduction in bioluminescence when compared to all other groups without TMZ. There was no statistical difference between the two groups receiving TMZ or KD-R + OAA + HBO_2_T (Figure 2B). Similarly, there was no statistical difference in average bioluminescence between genders in any study group (Supplementary Figure 2A). Immediately prior to termination of the first set of *in vivo* experiments, blood was collected from mice in all study groups. The GKI was determined for all mice based on the blood glucose and β-hydroxybutyrate values, as described in the materials and methods (Figure 2C). All groups under the KD-R had a GKI that was significantly lower than mice under the SD-UR. There was no difference in GKI values among groups under KD-R, regardless of the treatment administered.

**Figure 3:**
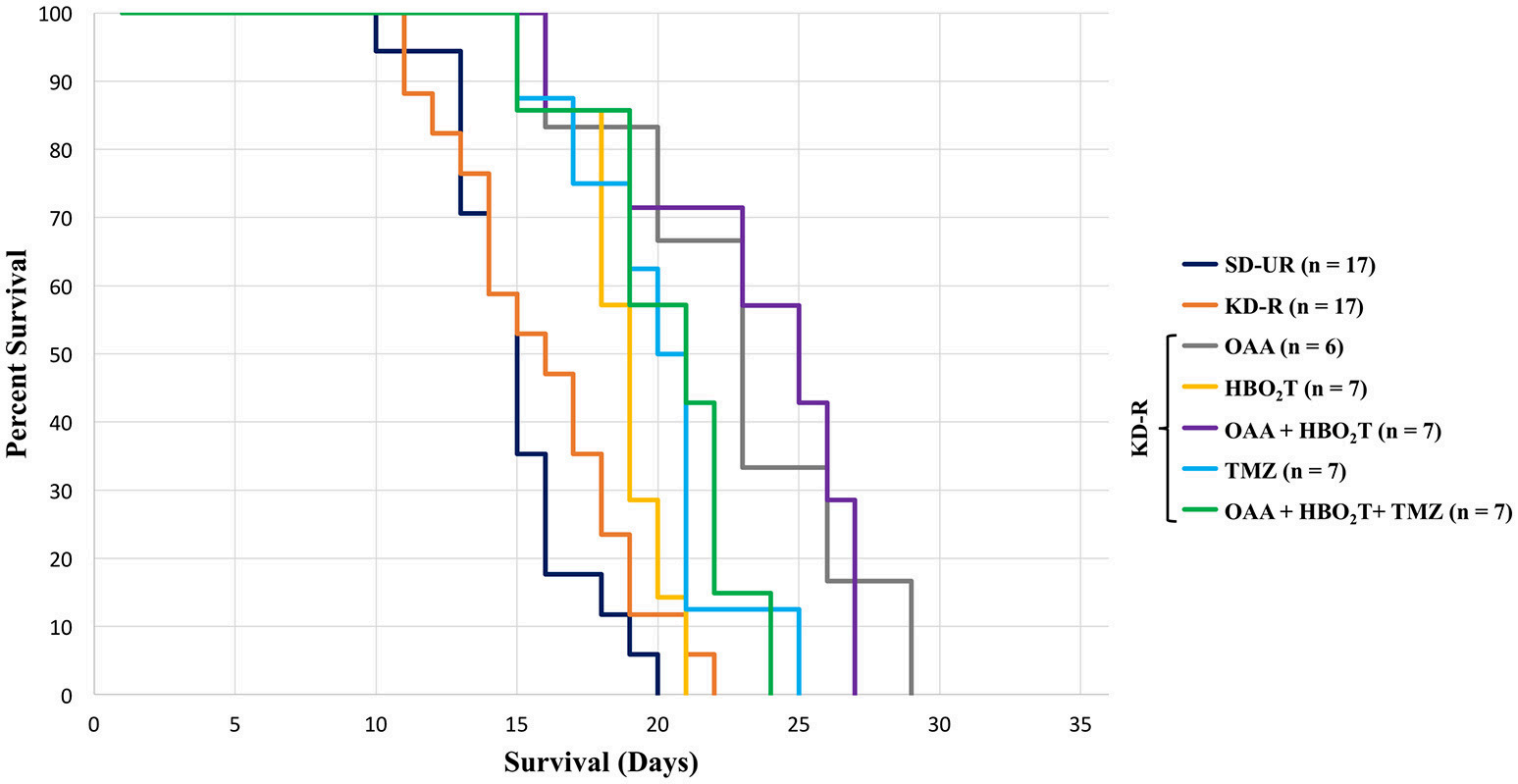
Influence of OAA, HBO_2_T, TMZ, and KD-R on average survival of tumor-bearing mice. A Kaplan Meier curve was used to determine mouse survival for each study group. The average survival for all study groups was calculated and the standard error of the mean was determined from these averages. Mice receiving TMZ had a lower survival than the groups receiving OAA without TMZ. Mice receiving OAA without TMZ were significantly different from SD-UR and KD-R (*P* < 0.05). Death was determined when mice became moribund and/or lost greater than 30% of their body weight.

Survival studies were also performed to determine if the therapeutic protocols used in the *in vivo* tumor progression studies also enhanced mouse survival. Mice were monitored daily and euthanized at the appearance of severe neurological damage from tumor growth or when they lost more than 30% of their starting body weight. SD-UR fed control mice reached morbidity at an average of 15 days post implantation (Figure 3). Survival was longest in the mice receiving OAA. The OAA alone did not significantly reduce tumor growth (Figure 2B), but enhanced the overall survival significantly in comparison to SD-UR and KD-R (Figure 3). A significant survival advantage was also found when OAA (2.0 mg/g) was combined with HBO_2_T under KD-R. TMZ treated mice reached morbidity at an average of 20 days due to potential drug toxicity, as indicated by significant body weight loss by day 11 and apparent lethargy. Though the brain tumor bioluminescence in the TMZ treated mice was lower than all other treated groups, the overall survival was diminished in comparison, potentially due to the toxic effects of TMZ (2). There was no statistical difference in survival between genders in any study group (Supplemental Figure 2B).

## Discussion

The goal of this study was to determine if a press-pulse metabolic therapy would be more effective in reducing growth of the VM-M3 murine GBM model than would treatment with single therapies. We found that tumor growth inhibition and mouse survival was better under a combination treatment of KD-R, OAA, and HBO_2_T than under single agent treatment. Although inclusion of TMZ in the therapeutic cocktail caused significant reduction in VM-M3 GBM growth, it also caused excessive body weight loss and reduced survival in comparison to other treatment groups. Potential gastrointestinal troubles following the administration of TMZ may have caused the reduced food consumption and the subsequent loss of body weight. However, it is possible, that further studies may find that lower dosages and modes of TMZ delivery could impact the toxicity, efficacy, and survival seen in mice receiving combinatorial therapy with TMZ. It is important to note that although average survival improved significantly for mice receiving OAA alone, no significant reduction was found for bioluminescent photon count in comparison to the KD-R alone. Also, KD-R alone did not significantly reduce tumor growth in comparison to SDUR, which can be explained by the necessity of available glutamine along with glucose in cancers, especially GBM (6, 26).

Similarly, we found no statistically significant difference in tumor growth or survival between the KD-R group and the mice receiving HBO_2_T. Although Poff AM, et al. reports a statistically significant difference between the same two study groups, those studies were performed on VM mice with the VM-M3 tumor implanted subcutaneously into the flank rather than orthotopically into the cerebral cortex. Also, Poff AM, et al. initiated the therapeutic regimen on the day of tumor inoculation, whereas our mice started receiving treatment on day 4 (35). Furthermore, throughout most of the survival study, the overall health and appearance of mice receiving OAA in combination with HBO_2_T was noticeably better than in mice that received OAA alone. This led to our conclusion that OAA alone may serve as a supplement for maintaining cancer patient health, while the combination of a KD-R, OAA, and HBO_2_T could be a potential anti-cancer therapy. As progression free survival and overall survival are key determinants of therapeutic success for cancer therapy, our findings showed that this proposed metabolic therapy without the inclusion of TMZ was beneficial to therapeutic success in this preclinical GBM model.

Although several studies have suggested that OAA participates in the deamination of glutamate into α-ketoglutarate, serves as a mitochondrial biogenesis enhancer, and may act as a calorie restriction mimetic, the mechanism by which OAA influences cancer progression remains unclear (24, 25, 27, 28). It did not appear from our studies that OAA acted as a calorie restriction mimetic as no statistically significant differences were observed in glucose or ketone levels between any of the study groups under KD-R. Further studies are needed to evaluate mechanisms that underlie the observed effect between KD-R, OAA, and HBO_2_T along with the potential ability for OAA to participate in the deamination of glutamate and to serve as a mitochondrial biogenesis enhancer. However, it is important to note the observation that OAA may induce cell death in the human hepatic carcinoma cell line HepG2 through induction of apoptosis and ROS accumulation (49).

The effect observed in the mouse groups receiving KD-R, OAA, and HBO_2_T could prove more efficacious if combined with inhibitors of glucose, glutamine, and autophagy. Indeed, a recent study showed that a press-pulse therapeutic strategy used with a modified standard of care significantly improved the outcome of a GBM patient (50). Further combinations of nontoxic metabolic treatments must be studied to enhance the survival of GBM patients while maximizing their long-term health and quality of life. This study is one example of nontoxic targeting of energy metabolism in a preclinical GBM murine model. It also provides evidence for the efficacy of combinatorial HBO_2_T, OAA, and the KD-R for managing growth and progression of the VM-M3/Fluc tumor.

## Data availability

The raw data supporting the conclusions of this manuscript will be made available by the authors, without undue reservation, to any qualified researcher.

## Author contributions

ZA, CD, PM, and TS designed the research and experimental design. ZA, CD, ML, and PM performed the research and gathered the data. ZA analyzed the data. ZA wrote the paper. All authors read and approved the final manuscript.

### Conflict of interest statement

The authors declare that the research was conducted in the absence of any commercial or financial relationships that could be construed as a potential conflict of interest. The reviewer BC and handling Editor declared their shared affiliation.

## Abbreviations

TMZ: Temozolomide
GBM: Glioblastoma multiforme
OAA: Oxaloacetate
KD-R: Ketogenic Diet Restricted
HBO_2_T: Hyperbaric Oxygen Therapy
Glu: Glutamate
ROS: Reactive Oxygen Species
SD-UR: Standard Diet-Unrestricted
DMSO: Dimethylsulfoxide
ATM: Standard Atmospheric Pressure
PBS: Phosphate Buffered Saline.
