# Supplementary material for: Nontoxic Targeting of Energy Metabolism in Preclinical VM-M3 Experimental Glioblastoma
Source: Front Nutr. 2018 Oct 5;5:91. doi: 10.3389/fnut.2018.00091 (PMC6186985; doi:10.3389/fnut.2018.00091)
Supplement: Supplementary file 1 [file Table_1.DOCX]

Supplementary Material

**Nontoxic targeting of energy metabolism in preclinical VM-M3 experimental glioblastoma**

Zachary M. Augur^1^, Catherine M. Doyle^1^, Mingyi Li^1^, Purna Mukherjee^1^, and Thomas N. Seyfried^1^

^1^Thomas N. Seyfried Laboratory, Biology Department, Boston College, Chestnut Hill, MA, USA

*** Correspondence:**

Thomas N. Seyfried

Thomas.seyfried@bc.edu

## Supplementary Figures

## Supplementary Figure 1. Effect of high and low dose OAA and TMZ under KD-R on VM-M3/Fluc brain tumor growth.

Whole brain *ex-vivo* bioluminescent data show that a dose effect was apparent between low and high dose OAA under HBO_2_T and KD-R. Mice receiving high dose OAA had a significantly greater therapeutic benefit (*) when compared to KD-R alone and low dose OAA (P < .05). A similarly apparent dose effect between high and low dose TMZ under KD-R was also observed via bioluminescent quantification. A significant therapeutic benefit (*) for mice receiving high dose TMZ was observed when compared to KD-R alone and low dose TMZ (P < .05). There was no statistical difference in average bioluminescence between genders in either dose study (p > .05)

## Supplementary Figure 2. Influence of KD-R, OAA, HBO_2_T, and TMZ on VM-M3/Fluc brain tumor growth and mouse survival in male and female mice.

**(A)** Whole brain *ex-vivo* bioluminescent data disaggregated by gender is shown in the figure. The bioluminescent values for male and female mice in each study group were combined for Figure 2B. There was no statistically significant difference between gender for any study group (p > .05). **(B)** The average survival for all study groups was calculated based on gender and the standard error of the mean was determined from these averages. There was no statistically significant difference between genders in any study group (p > .05). Survival data for both male and female mice were combined for the Kaplan Meier curve in Figure 3.
