# Supplementary figures and images for: Nontoxic Targeting of Energy Metabolism in Preclinical VM-M3 Experimental Glioblastoma
Source: Front Nutr. 2018 Oct 5;5:91. doi: 10.3389/fnut.2018.00091 (PMC6186985; doi:10.3389/fnut.2018.00091)

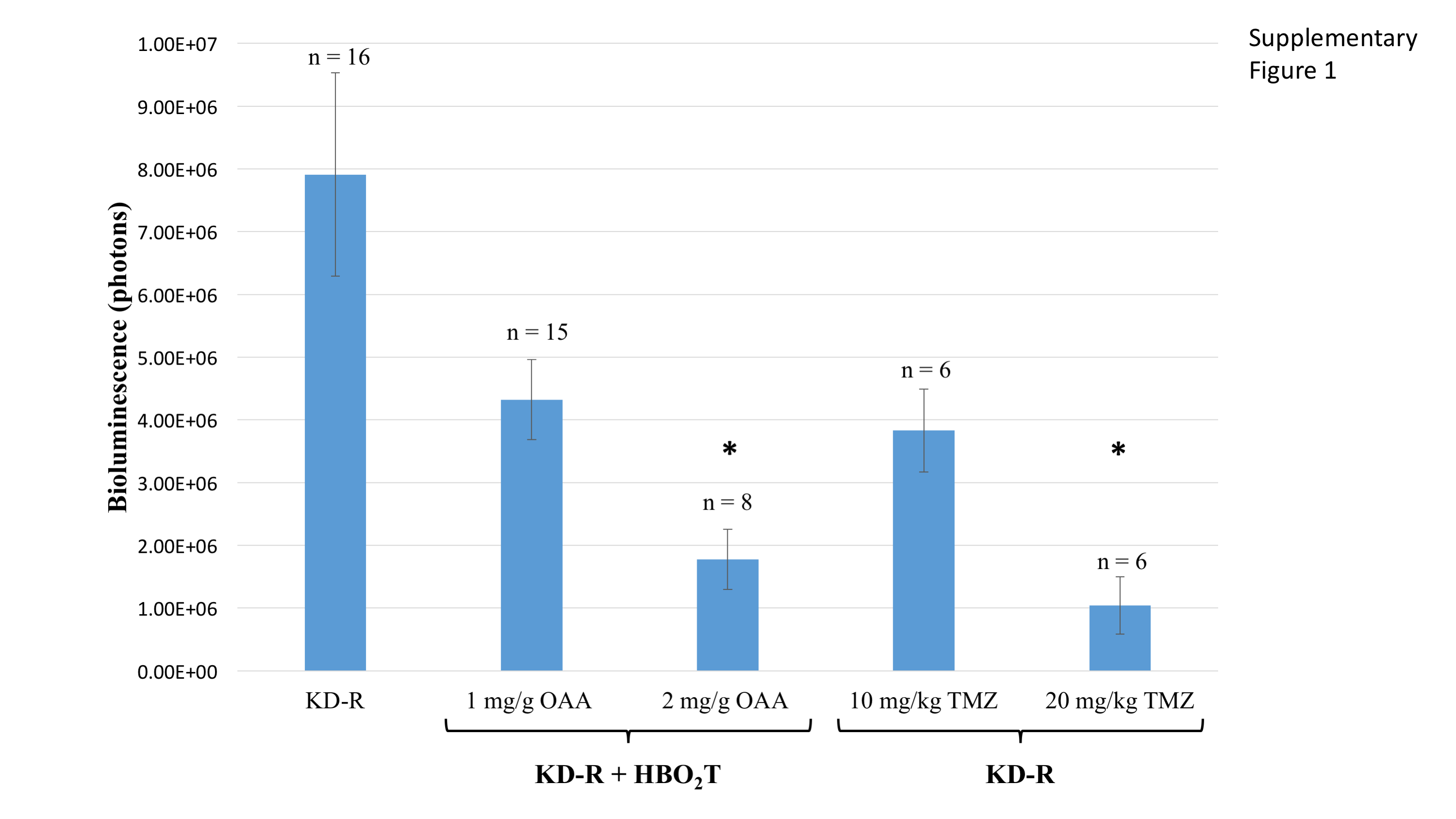

Supplement: Supplementary file 2 [file Image_1.TIFF]

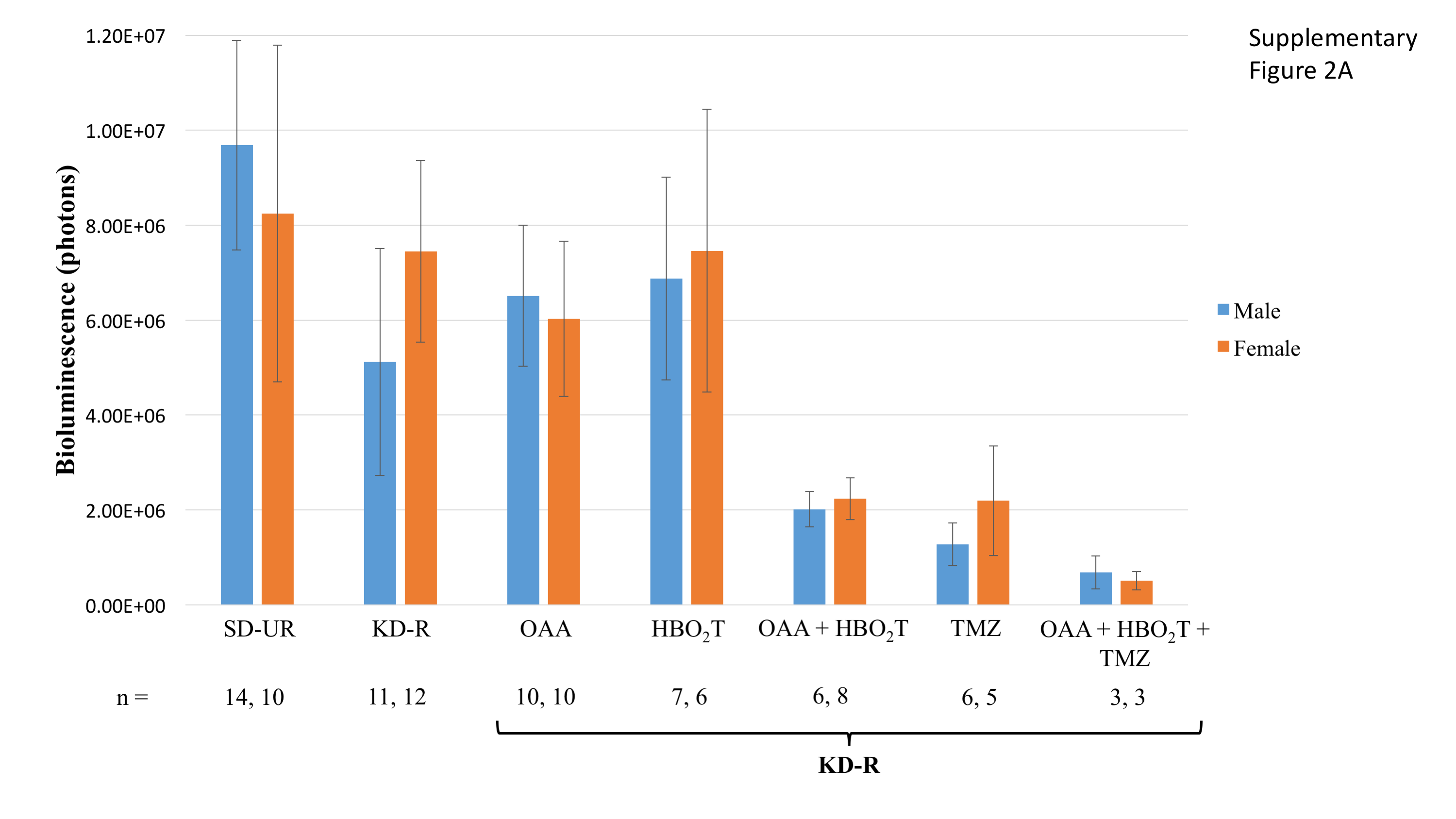

Supplement: Supplementary file 3 [file Image_2.TIFF]

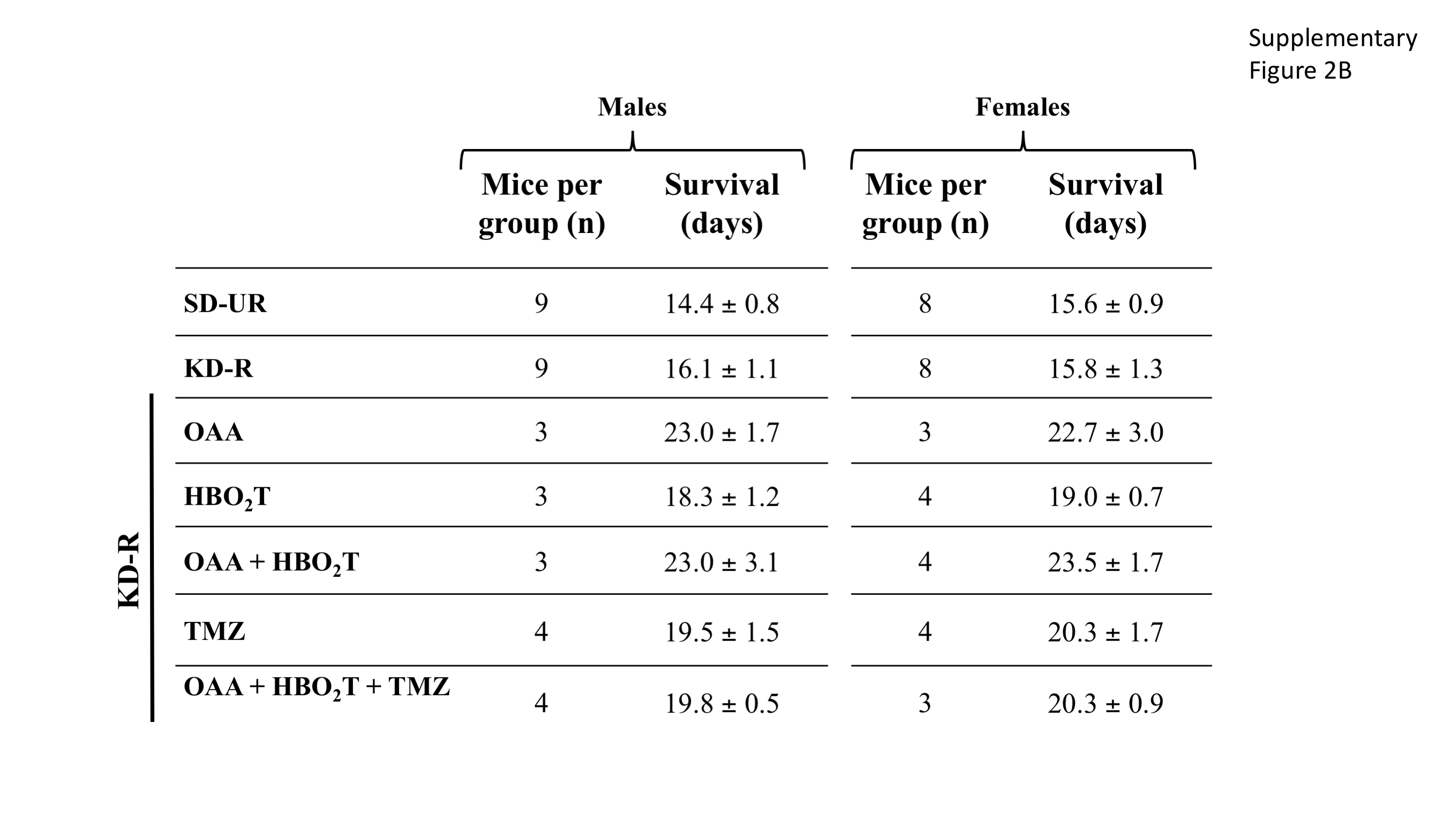

Supplement: Supplementary file 4 [file Image_3.TIFF]
